# Prevalence of high blood pressure and association with obesity in Spanish schoolchildren aged 4–6 years old

**DOI:** 10.1371/journal.pone.0170926

**Published:** 2017-01-31

**Authors:** Noelia Martín-Espinosa, Ana Díez-Fernández, Mairena Sánchez-López, Irene Rivero-Merino, Lidia Lucas-De La Cruz, Montserrat Solera-Martínez, Vicente Martínez-Vizcaíno, Movi-Kids group

**Affiliations:** 1 Universidad de Castilla-La Mancha, Faculty of Nursing, Toledo, Spain; 2 Universidad de Castilla-La Mancha, Health and Social Research Centre, Cuenca, Spain; 3 Universidad de Castilla-La Mancha, Faculty of Nursing, Cuenca, Spain; 4 Universidad de Castilla-La Mancha, Faculty of Education, Ciudad Real, Spain; 5 Hospital General Universitario, Ciudad Real, Spain; 6 Facultad de Ciencias de la Salud, Universidad Autónoma de Chile, Talca, Chile; Shanghai Institute of Hypertension, CHINA

## Abstract

**Background:**

The prevalence of high blood pressure in children is increasing worldwide, largely, but not entirely, driven by the concurrent childhood obesity epidemic. The aims of this study were to examine the prevalence of prehypertension and hypertension in 4-to-6-year-old Spanish schoolchildren, and to evaluate the association between different blood pressure (BP) components with different adiposity indicators.

**Methods:**

Cross-sectional study including a sample of 1.604 schoolchildren aged 4-to-6-years belonging to 21 schools from the provinces of Ciudad Real and Cuenca, Spain. We measured height, weight, body mass index (BMI), fat mass percentage (%FM), triceps skinfold thickness (TST), waist circumference (WC), systolic and diastolic BP, mean arterial pressure and pulse pressure.

**Results:**

The estimates of prevalence of prehypertension and hypertension were 12.3% and 18.2%, respectively. In both sexes, adiposity indicators were positively and significantly associated with all BP components (p<0.001), thus schoolchildren in the higher adiposity categories had significantly higher BP levels (p<0.001).

**Conclusions:**

Our results show a high prevalence of high blood pressure in Spanish children. Moreover, high levels of adiposity are associated with high blood pressure in early childhood, which support that it could be related to cardiovascular risk later in life.

## Introduction

Over the last decade, epidemiologic studies have reported an increase in children’s blood pressure (BP) levels [1], as well as in the prevalence of prehypertension and hypertension [2**–**6] largely, but not entirely, driven by a concurrent increase in childhood obesity. This fact has been accompanied by an increased recognition of the importance of BP measurements in children. However, the importance of monitoring BP levels in the pediatric age goes beyond its association with obesity because it has been consistently reported that, independently of body mass index (BMI), BP levels track from childhood to adulthood [7, 8], and that BP levels in childhood predict young adult cardiovascular risk [9]. Despite the extensively reported increase in the prevalence of high BP in the pediatric population worldwide, only a few studies have examined BP estimates in Spanish children, reporting that this prevalence ranged from 1.7% to 3.2% [10, 11].

The prevalence of overweight/obesity is above 35% in Spanish children [12**–**14]. A recent study conducted in 4-to-6 years old children from the Castilla-La Mancha region (Spain) reported an overweight/obesity prevalence of 20.4% [15]. Elevated BP levels should be associated with this high obesity prevalence estimation, but studies analyzing the association between indicators of adiposity and BP are scarce in Spanish children [10, 11] and none have been conducted in children ≤ 6 years old.

Traditionally, BP measurements only include systolic blood pressure (SBP) and diastolic blood pressure (DBP) values, but other indices, such as mean arterial blood pressure (MAP) or pulse pressure (PP), have also been shown to be independent predictors of cardiovascular events in both normotensive and hypertensive adult individuals [16, 17].

Because the current estimates of overweight/obesity prevalence in Spanish children are one of the highest in the world, estimates of high blood pressure prevalence in Spanish children might represent an indirect indicator of the impact of the obesity epidemic on BP levels. Thus, the aims of this study were to examine in 4-to-6 years old Spanish schoolchildren: 1) the prevalence of prehypertension and hypertension and 2) the association between adiposity indicators (BMI, %FM, TST and WC) with traditional (SBP, DBP) and alternative (MAP, PP) blood pressure components.

## Methods

### Study design and population

This was a cross-sectional analysis of baseline data (collected September–November 2013) from a cross-over randomized cluster trial (NCT01971840) aimed at assessing the effectiveness of a multidimensional physical activity intervention (Movi-Kids) for preventing obesity, improving fitness and reducing cardiovascular risk in schoolchildren belonging in the third grade of preschool and the first grade of primary school (aged 4-to-6 years) [18]. The Movi-Kids study included 1.604 schoolchildren from 21 primary schools (19 public, two private) from different towns of Cuenca and Ciudad Real provinces, Castilla-La Mancha region, Spain. The Clinical Research Ethics Committee of the “Virgen de la Luz” Hospital in Cuenca approved the study protocol. After obtaining the approval of the director and school committee of each school, we sent a letter to the parents of all children inviting them to a meeting in which the study objectives were outlined and a written consent for their children's participation was requested. Then, we held informative talks class by class, in which the schoolchildren were asked to participate in the study.

### Anthropometrics and blood pressure measurements

Measurement procedures have been extensively described elsewhere [18]. To minimize inter-observer variability, the variables were measured in each school using standardized conditions by trained researches.

Weight, height and %FM were measured twice with the child barefoot and in light clothing. Weight was measured with a scale (Seca^®^ 861, Vogel and Halke, Hamburg, Germany) and height was measured using a wall stadiometer (Seca^®^ 222, Vogel and Halke, Hamburg, Germany) with the child upright and with the sagittal midline touching the backboard. The mean of the two measurements of weight and height was used to calculate BMI (kg/m^2^). %FM was estimated using an eight-electrode Tanita^®^ Segmental-418 bioimpedance analysis system (Tanita Corp. Tokyo, Japan). Two readings were obtained in the morning, under controlled temperature and humidity conditions, with the child being barefoot, fasting and after urination and 15 minutes of rest. Triceps skinfold thickness (TST) was measured three times at the triceps using a Holtain Ltd. caliber (0.2mm accuracy and consistent 10g/mm2 pressure between valves). Waist circumference (WC) was calculated as the average of three measurements using a flexible tape at the midpoint between the last rib and the iliac crest at the end of a normal breathe (exhalation).

SBP and DBP were measured twice, taken at an interval of five minutes, with the subject resting for at least five minutes before the first measurement, using an OMRON-M5-I automatic tensiometer (Omron Healthcare Europe BV, Hoofddorp, Netherlands), in a quiet, calm environment, with the child seated and the right arm placed in a semi-flexed position at heart level and choosing the most appropriate size of the cuff according to recommendations of the National High Blood Pressure Education Program Working Group on High Blood Pressure in Children and Adolescents [19]. The mean of the two readings was considered for the analysis. The MAP and the PP were calculated as follows: MAP = DBP + (0.333 x (SBP-DBP)), and PP = SBP-DBP. According to the National High Blood Pressure Education Program Working Group on High Blood Pressure in Children and Adolescents [19], categories of high BP in the schoolchildren were established according to the percentile for sex, age, and height as follows: prehypertension, when the average of the SBP or DBP was greater than or equal to the 90^th^ percentile; and hypertension, when the average of the SBP or DBP was greater than or equal to the 95th percentile. Into the latter, we distinguish: stage 1 (from the 95th percentile to the 99th percentile plus 5 mm Hg) and stage 2 (>99th percentile plus 5 mm Hg).

### Statistical analysis

The distribution of continuous variables was checked for normality using both graphical (normal probability plot) and statistical (Kolmogorov—Smirnov test) procedures. All variables had a normal distribution, thus we used parametric test in the analysis. Anthropometrical and BP variables were presented as mean and standard deviation (SD). Sex differences on the quantitative variables were tested using a Student T-test.

Partial correlation coefficients were estimated to examine the relationship between each BP component (SBP, DBP, MAP and PP) and indicators of adiposity (BMI, %FM, TST and WC), controlling for age, by sex.

We categorized %FM, TST and WC as low (first quartile), medium (second and third quartiles) and high (fourth quartile). Children were classified as underweight, normal weight, overweight and obese according to the BMI cut-offs proposed by Cole and Lobstein [20].

ANCOVA models were used to assess mean differences in each component of BP (SBP, DBP, MAP and PP) among BMI, %FM, WC and TST categories controlling for age in the total sample and also separately by sex. Pairwise *post hoc* hypotheses were tested using the Bonferroni correction for multiple comparisons.

All statistical analyses were performed using the IBM SPSS 22 Statistic software; the criterion for statistical significance was set at p ≤ 0.05.

## Results

Of the 2.407 schoolchildren invited to participate in the study, 1.604 (66.63%) agreed to participate. Out of these, 788 were girls (49.10%). No significant differences were observed between the mean age of the girls and boys. Summary statistics of all variables are shown in Table 1.

**Table 1:** Characteristics of the study sample.

	Total (n = 1604)	Boys (n = 816)	Girls (n = 788)	P value
Age (years)	5.34 ± 0.60	5.32 ± 0.60	5.37 ± 0.62	0.135
Weight (Kg)	21.39 ± 4.77	21.66 ± 4.85	21.11 ± 4.68	**0.022**
Height (cm)	115.48 ± 6.07	115.93 ± 6.08	115.01 ± 6.03	**0.002**
BMI (Kg/m2)	15.89 ± 2.47	15.97 ± 2.45	15.82 ± 2.48	0.243
% FM	20.1 ± 5.85	20.01 ± 5.23	20.37 ± 6.42	0.226
TST (mm)	11.03 ± 4.64	12.53 ± 4.59	11.77 ± 4.67	**<0.001**
WC (cm)	56.10 ± 6.1	55.32 ± 6.22	55.72 ± 6.1	**0.012**
SBP (mm Hg)	102.14 ± 10.16	102.66 ± 10.32	101.61 ± 9.98	**<0.001**
DBP (mm Hg)	62.28 ± 8.52	61.68 ± 8.26	62.89 ± 8.74	**0.005**
MAP (mm Hg)	75.55 ± 8.36	75.33 ± 8.21	75.78 ± 8.50	0.279
PP (mm Hg)	39.86 ± 7.64	40.97 ± 7.8	38.72 ±7.3	**<0.001**

Data are presented by mean ± SD. Abbreviations: BMI = body mass index; FM = fat mass; TST = triceps skinfold thickness; WC = waist circumference; SBP = systolic blood pressure; DBP = diastolic blood pressure; MAP = mean arterial pressure (DBP + {0.333 x (SBP—DBP)}); PP = pulse pressure (SBP-DBP). In bold when p value ≤ 0.05

Partial correlation coefficients between BP and adiposity indicators, controlling for age, are shown in Table 2. All variables were positively and significantly associated with BP components included in the study in both sexes (p<0.001).

**Table 2:** Partial correlations coefficients (*r*) of systolic blood pressure, diastolic blood pressure, mean arterial pressure and pulse pressure with BMI, %fat mass, WC and TST controlling for age.

		BMI	%FM	WC	TST
**SBP**	Total	0.354	0.331	0.345	0.231
	Boys	0.354	0.315	0.342	0.259
	Girls	0.352	0.353	0.345	0.226
**DBP**	Total	0.227	0.235	0.220	0.156
	Boys	0.231	0.226	0.227	0.156
	Girls	0.232	0.240	0.229	0.140
**MAP**	Total	0.297	0.293	0.289	0.199
	Boys	0.302	0.282	0.294	0.212
	Girls	0.296	0.303	0.292	0.184
**PP**	Total	0.215	0.175	0.211	0.132
	Boys	0.225	0.177	0.213	0.178
	Girls	0.199	0.191	0.192	0.139

Abbreviations: BMI = body mass index; FM = % fat mass; WC = waist circumference; TST = triceps skinfold thickness; SBP = systolic blood pressure; DBP = diastolic blood pressure; MAP = mean arterial pressure (DBP + {0.333 x (SBP—DBP)}); PP = pulse pressure (SBP-DBP). All coefficients were significant (p<0.001).

**Fig 1:**
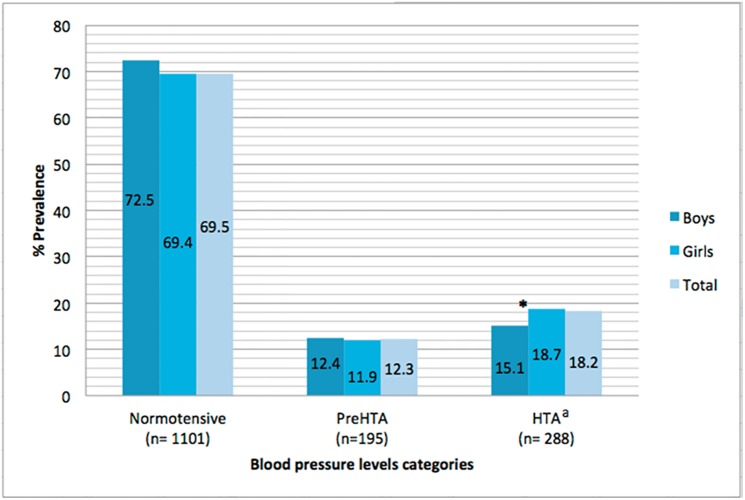
Prevalence of normotensive, prehypertension and hypertension in children, by sex and in the total sample. Abbreviations: PreHTA = prehypertension; HTA = hypertension. ^a^HTA includes stages 1 and 2. *Indicates sex differences in the prevalence of HTA category (p<0.05).

Fig 1 depicts the prevalence of high BP by sex and age. Estimates of prehypertension and hypertension were 12.3% and 18.2%, respectively. No statistically significant differences were found between the estimates of prehypertension prevalence by sex groups, but girls had a significantly higher prevalence of hypertension than boys (p<0.05).

Mean differences in all BP components (SBP, DBP, MAP, PP) by categories of BMI and %FM, controlling for age, are shown in Table 3. In the total sample, children in the higher adiposity categories had significantly higher BP levels (p<0.001). Also significant higher BP mean levels by adiposity categories were found when TST and WC were included as fixed factors (p<0.001) (data not shown). All pairwise Bonferroni *post hoc* tests were statistically significant. Similar results were also found when data were analyzed separately by sex (p<0.001) (data not shown).

**Table 3:** Mean differences in blood pressure parameters according to adiposity categories in total sample, controlling for age.

**BODY MASS INDEX**							
	**UW** n = 324	**NW** n = 930		**OV** n = 188		**OB** n = 134	**p**
**SBP**	98.14 ± 9.42	101.43 ± 9.18		106.37 ± 9.76		110.95 ± 11.73	**<0.001**
**DBP**	60.84 ± 8.03^a^	61.58 ± 8.35		64.54 ± 8.56		67.43 ± 0.47	**<0.001**
**MAP**	73.26 ± 7.83	74.85 ± 7.96		78.47 ± 8.1		81.92 ± 0.67	**<0.001**
**PP**	37.3 ± 7.11	39.84 ± 7.12		41.82 ± 8.22^b^		43.52 ± 9.14	**<0.001**
**% FAT MASS**							
	**Low** n = 394		**Medium** n = 788		**High** n = 394		**p**
**SBP**	98.63 ± 8.98		101.5 ± 9.4		106.97 ± 10.92		**<0.001**
**DBP**	60.55 ± 8.11		61.59 ± 8.22		65.38 ± 8.75		**<0.001**
**MAP**	73.23 ± 7.75		74.88 ± 7.92		79.23 ± 8.63		**<0.001**
**PP**	38.07 ± 6.93		39.91 ± 7.27		41.58 ± 8.58		**<0.001**

Data are presented as mean ± SD. Abbreviations: SBP = systolic blood pressure; DBP = diastolic blood pressure; MAP = mean arterial pressure (DBP + {0.333 x (SBP—DBP)}); PP = pulse pressure (SBP-DBP). Categories of BMI are Underweight (UW), Normal Weight (NW), Overweight (OV) and Obesity (OB) according to gender-and-age-specific cut-offs defined by Cole and Lobstein (20). Categories of fat mass are Low, Medium, and High, representing the 1st, 2nd and 3rd and 4th quartiles. All post-hoc hypothesis tests using the Bonferroni correction for multiple comparisons were statistically significant (p value <0.001) except for those with superscript letters:

^a^ UW<NW and

^b^ OV<OB.

## Discussion

Studies estimating prevalence of high blood pressure in children ≤ 6 years old are scarce across the world, and none have been performed in Spain. This study shows that high blood pressure prevalence in children aged 4-to-6 years from Castilla-La Mancha, Spain, was 27.5% and 30.6% in boys and girls, respectively. Furthermore, the prevalence of prehypertension and hypertension in the total sample was 12.3% and 18.2%, respectively. Likewise, a positive relationship between adiposity categories and BP levels has been found.

A significant variability of hypertension (≥95^th^ percentile) prevalence has been reported in different population-based studies in the same age-range groups of children across the world; some of them were similar to our results: 23% in China [21] and 19.9% in Brazilian children [22]. Lower percentages were reported in Sydney, 13.7% [23], Seychelles, 12% [24] and 6.4% in Minnesota and California [25].

Potential reasons that would be argue to explain this variability in blood pressure levels across countries include differences in the procedures used for the measurement of BP across these studies, and dissimilarities in the trends of obesity and samples which include children of different ethnicities.

In Spain, no specific studies about the prevalence of hypertension in children aged less than 7 years, using the classification of BP established by the 4th report have been conducted so far. Three studies, using the Ricardin study criteria, reported high blood pressure prevalence estimates [26], ranging from 1.7% to 4.5% in children aged 6–12 [10, 11, 27]. We cannot compare our results with those studies, since the Ricardin study considers only cut-off point criteria in children aged 6 to 18 years, and the age of most participants in our study is out of this age-range.

The relationship between adiposity with different BP components in children has been shown in several studies. Eisenmann et al. reported that BMI, WC, sum of skinfolds and %FM (measured via dual energy X-ray absorptiometry) were moderately and positively correlated with SBP, DBP and MAP [28], as found in other studies in which BMI [27, 29] and TST [30] were associated with SBP and DBP. Our results, in line with other studies suggest that, overall, the intensity of the association of different indicators of adiposity is similar with SBP and physiologic BP components (MAP, PP) [31, 32], supporting that children with more adiposity are more likely to have higher risk of hypertension regardless the BP component used.

Our findings also suggest that children in the higher categories of BMI and %FM have higher levels of SBP, DBP, MAP and PP in both boys and girls, as reported in other studies in the same age group and different ethnicities [33**–**37]. However, findings of longitudinal studies are discordant since meanwhile some authors concluded that an increase in the rate of obesity partially explained the rise of high blood pressure [38, 39], others found that the prevalence of elevated blood pressure decreased while the obesity prevalence increased [40, 41], supporting the idea that children with high BMI levels at such early age are not at high probability of becoming hypertensive or have high blood pressure during adolescence [41]. Therefore, other factors, like physical fitness or changes in the diet [42, 43] may be influencing this longitudinal relation.

It has been suggested that cardiovascular risk attributable to hemodynamic factors may be assessed more accurately considering physiological (MAP, PP) rather than traditional (SBP, DBP) components of BP, using PP as an indicator of large artery stiffness (pulsatile load) and MAP as an indicator of peripheral resistance and cardiac output (steady flow load) [44]. It has been hypothesized that excess body weight may be responsible for a mismatch between pulsatile blood flow and aortic size and between cardiac output and peripheral resistance, and therefore physiologic indicators like PP and MAP might be closely related to adiposity indicators compared to traditional BP measures [16, 44]. In relation with the use of PP as a predictor of cardiovascular disease, the NHANES III found that there were more obese children in the higher PP quartile compared with normal weight children, independent of age, ethnicity and gender [45], and our results are in agreement with this tendency.

The association between overweight/obesity with physiologic BP components (MAP and PP) at such an early ages might have an impact on the large arteries, which would need to adjust their size to a higher blood volume, increasing their diameter. This enlarged arterial stiffness might determine higher risk of future hypertension and cardiovascular disease, but longitudinal studies are needed to assess this association in adulthood [44].

This study has some limitations that should be noted. First, the cross-sectional design which prevents us from making cause-effect inferences. Second, potential overestimation of the prevalence of high BP due to the determination of BP in a single occasion, compared to others studies that reported two more screenings of BP in those individuals classified as prehypertensive and hypertensive [22, 46]. According to the Fourth Report, the recommended measurement of BP should be performed using an auscultatory method [19], but in the present study BP measurements were obtained using an automatic oscillometric monitor validated for children. Unfortunately, we were not able to do it in the current study because the measurements were performed in school settings in a single day, so we selected automatic oscillometric monitor to avoid inter-observer variability, since is usually difficult to distinguish between 4th and 5th Korotkoff sound in young children, even for trained nurses. Finally, taking measurements in schools setting could also influence BP values because of the difficulties that sometimes exist in maintaining a quiet and calm environment.

## Conclusion

Our data show a high prevalence of prehypertension and hypertension in Spanish children younger than 6 years. These findings are important from a clinical and public health point of view because they support the idea that early detection of a prehypertensive and hypertensive status in young children may help to prevent the cardiovascular disease in adulthood in Spanish population.
